# Microbial diversity and potential pathogens associated with the plastisphere on beaches of Rio de Janeiro, Brazil

**DOI:** 10.1007/s42770-026-01880-5

**Published:** 2026-03-09

**Authors:** Luana Gomes Morais, Henrique Fragoso dos Santos, Douglas Alfradique Monteiro, Marina Montenegro Mascarenhas, Helena Antunes Portela, Mônica Regina da Costa Marques, Fábio Vieira de Araújo

**Affiliations:** 1 Inst. de Biologia, Universidade Federal Fluminense, Rua Professor Marcos Waldemar de Freitas Reis, Niterói, RJ 24210-201 Brazil; 2 Faculdade de Geologia, Universidade do Estado do Rio de Janeiro, Rua São Francisco Xavier 524, Maracanã, Rio de Janeiro, RJ 20550-900 Brazil; 3 Instituto de Química, Universidade do Estado do Rio de Janeiro, Rua São Francisco Xavier 524, Maracanã, Rio de Janeiro, RJ 20550-900 Brazil; 4 Faculdade de Formação de Professores, Universidade do Estado do Rio de Janeiro, Rua Francisco Portela 1470, Patronato, São Gonçalo, RJ 24435-005 Brazil

**Keywords:** Marine litter, Human pathogens, Marine organisms pathogens, Plastic-degrading bacteria

## Abstract

Marine pollution caused by plastic waste is a global issue. Improperly discarded plastics often end up in seas and oceans, where they become colonized by a microbial biofilm known as the Plastisphere. In this environment, especially in places where sewage is discharged untreated into water bodies, pathogenic microorganisms and those carrying antimicrobial resistance genes (ARGs) can settle and spread over long distances. To assess the presence of these microorganisms and examine the impact of substrate type on the formation of these communities, plastic waste samples were collected from three beaches in Rio de Janeiro, Brazil. The plastics were identified as polypropylene (PP) and polyethylene (PE) using FTIR analysis, and the microbial biofilm attached to them was studied through 16 S rRNA gene sequencing. The results revealed the presence of bacteria potentially pathogenic to humans and marine life, as well as plastic-degrading species in the biofilms of the analyzed samples. Although the communities present in the different polymers in this study clustered separately, our results did not show specific associations between polymer types and the observed microbial communities. The spread of these plastics in the ocean poses risks to human health and marine biodiversity.

## Introduction

Global plastic production is projected to reach 460 million tons by 2030 [1], with approximately 22% of this total being improperly discarded [2], contributing to the increasing accumulation of plastic waste in marine environments. Jacquin et al. [3] report that between 4.8 and 12.7 million tons of plastic enter the ocean annually due to improper urban waste disposal, coastal tourism, and maritime activities. This pollution has become a global issue, significantly impacting coastal and marine environments [4].

Once in the ocean, these pollutants rapidly disperse over long distances due to ocean currents and winds, facilitating their deposition in coastal areas [5, 6]. Plastic in the marine environment poses numerous problems for organisms, including entanglement and accidental ingestion, which can cause illness and death in animals such as turtles, birds, fish, and marine mammals [7, 8].

Due to characteristics such as longevity, resilience, and hydrophobicity, drifting plastic debris serves as a persistent artificial surface for microbial colonization [9], and surface roughness and the size of the debris can accelerate this process [10]. This colonization leads to the formation of a biofilm, known as the Plastisphere [11].

The formation of biofilms on the surface of plastics is important for the degradation process [12], as it can protect the material from UV radiation, thereby reducing the direct action of photocatalysis or indirectly by decreasing its buoyancy [13], which increases its persistence in marine debris. On the other hand, studies show that microorganisms can accelerate the degradation of these materials [9].

The Plastisphere is also responsible for the dispersion of microorganisms, some of which are potentially pathogenic [14], and others that carry antimicrobial resistance genes [15, 16] throughout the ocean. The presence of these microorganisms in biofilms formed on plastic debris in the ocean enhances the role of water as a vehicle for the transmission of various diseases. In this new environment, microorganisms may encounter conditions that increase their viability and promote gene transfer mechanisms, thereby raising the risk for a significant portion of the population that relies on coastal environments for recreation and subsistence [4].

Moreover, microorganisms present in the Plastisphere represent a key category of life to understanding and mitigating the potential adverse effects of plastics due to their role in driving the overall functioning of the marine biosphere and as possible mediators of the biodegradation of associated plastic additives, contaminants, or even the plastics themselves [17]. Therefore, examining the formation, structure, and functions of biofilms related to this type of waste is crucial to inform management strategies aimed at preserving the ecological integrity of marine ecosystems.

Globally, research has focused on the composition, quantification, and distribution of marine plastic waste; however, much less attention has been given to the microbial communities colonizing these materials, particularly in tropical environments. These regions often encompass developing countries with limited sanitation infrastructure, where pathogenic microorganisms and carriers of antimicrobial resistance genes—once present in waters receiving untreated effluents—can be transported by plastic waste to previously unpolluted areas [18, 19].

Thus, this study aimed to characterize the bacterial community in the Plastisphere of plastic waste with distinct polymer compositions found on urban beaches in Rio de Janeiro. The study tested the following hypotheses: (i) potentially pathogenic microorganisms and plastic-degrading species are present in the Plastisphere of plastics with distinct polymer compositions; (ii) the polymer composition of plastic waste influences the composition of the Plastisphere.

Our study holds significant value as it sheds light on microbial communities associated with plastic waste in a region previously unexplored in this context. While several studies have examined the Plastisphere in temperate environments, little is known about microbial communities colonizing plastics in tropical urban bays subjected to severe sewage contamination, such as Guanabara Bay, one of the most polluted coastal systems in South America.

## Methodology

### Study areas

**Fig. 1:**
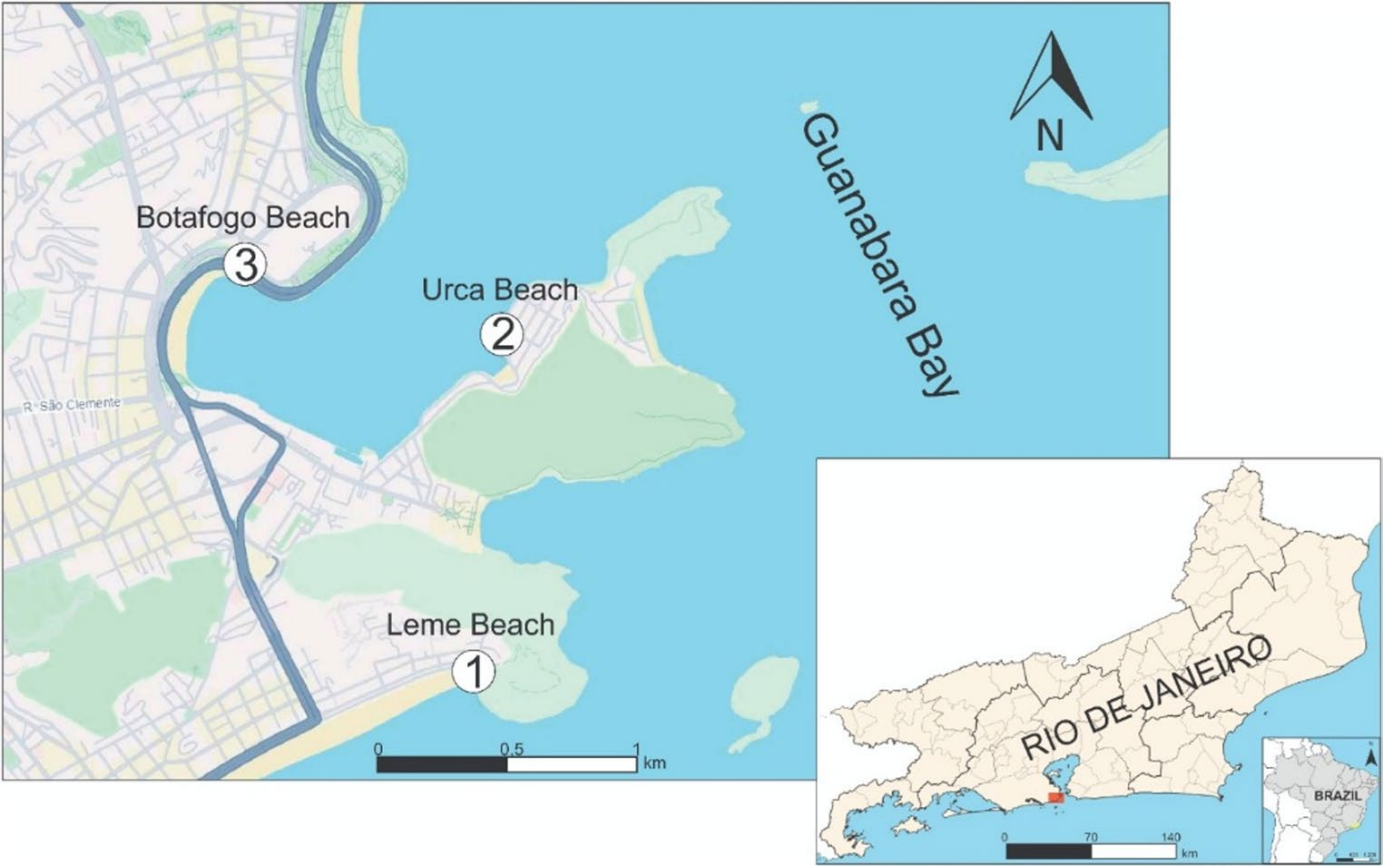
Map showing the collection sites: Leme (thermotolerant coliform < 1,000/100mL), Urca (thermotolerant coliform ≥ 1,000–10,000/100 mL), and Botafogo (thermotolerant coliform > 10,000/100 mL) beaches in Rio de Janeiro (RJ), Brazil. The sampling sites were designated as 1 (Leme Beach), 2 (Urca Beach), and 3 (Botafogo Beach)

Rio de Janeiro, considered the largest coastal city in Latin America, with a population of 6,750,000 inhabitants, is renowned worldwide for its natural landscapes and is a highly sought-after tourist destination. Despite this, some municipalities in their metropolitan region lack sanitation infrastructure, leading to pollution of the waters of Guanabara Bay, which receives effluent from several of these municipalities. The selected study sites include the beaches of Leme, Urca, and Botafogo, located in the urban area of Rio de Janeiro, RJ (Fig. 1). These beaches were chosen as representative sites due to their distinct pollution gradients.

Leme is an oceanic beach near the entrance to Guanabara Bay, offering favorable conditions for swimming nearly all the time (thermotolerant coliform levels below 1,000/100mL), according to INEA [20]. It has a high flow of bathers and is frequented by various vendors along its sands. Urca and Botafogo beaches, which exhibit thermotolerant coliform counts of 1,000–10,000/100 mL and > 10,000/100 mL, respectively, are in Guanabara Bay, a region known for its high levels of pollution. Although both beaches have calm waters, Botafogo exhibits poorer water quality than Urca, as it is in a more sheltered area with reduced water circulation. While the water at Urca is not always suitable for swimming, it is frequented by bathers, unlike Botafogo beach [20]. Despite this, both beaches serve as locations for recreational activities on their sands.

### Sampling

**Fig. 2:**
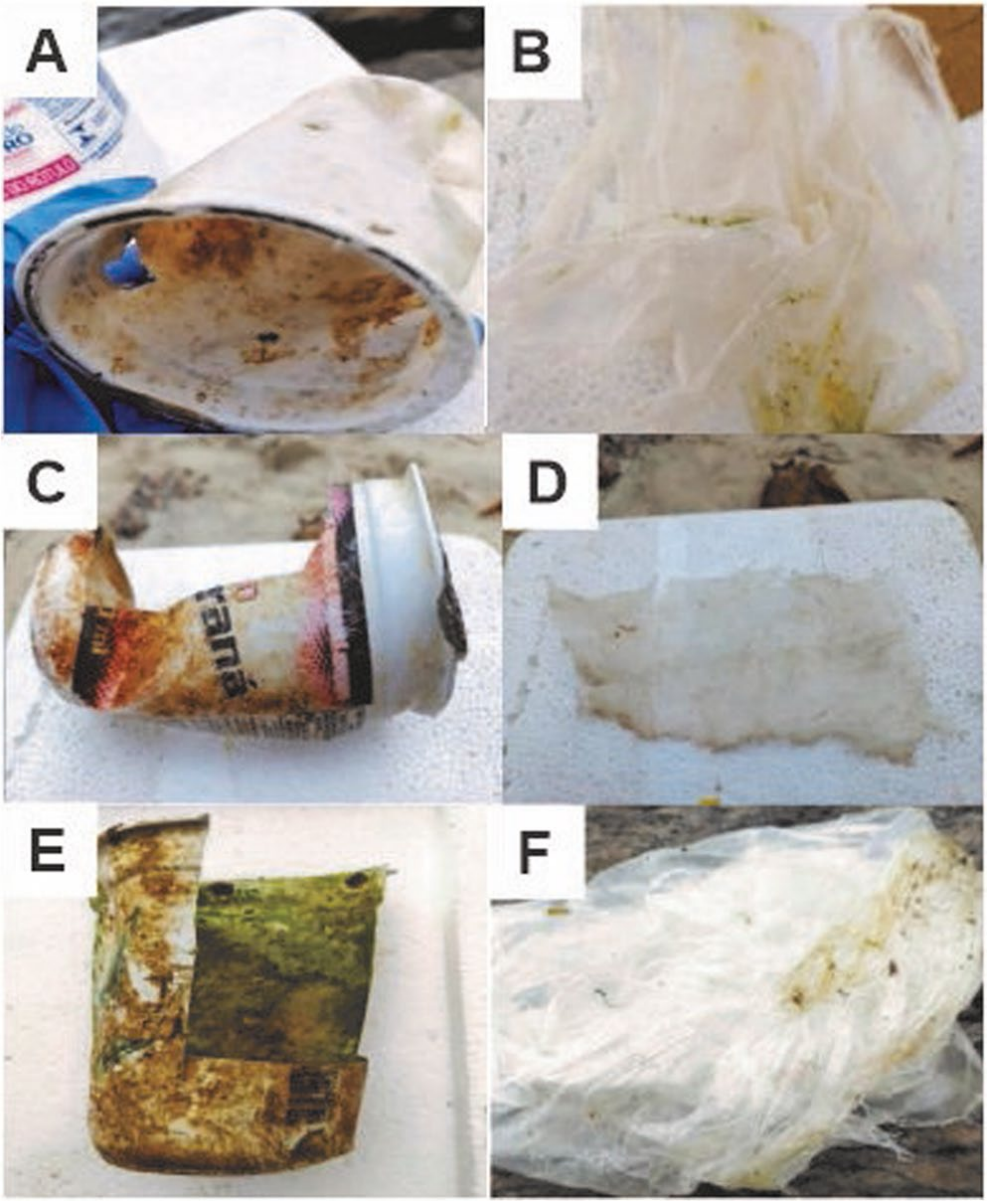
Plastic cups (**A**, **C**, and **E**) and plastic bags (**B**, **D**, and **F**) collected at Leme Beach (**A**, **B**), Urca Beach (**C**, **D**), and Botafogo Beach (**E**, **F**)

The collections were carried out on March 28, 2022, under moderate winds and no precipitation, according to the National Institute of Meteorology (INMET). The collection began at Leme Beach at 14:00 (Latitude: −22.9635213, Longitude: −43.1649918), followed by Urca Beach at 15:30 (Latitude: −22.9471088, Longitude: −43.1634273), and concluded at Botafogo Beach around 16:30 (Latitude: −22.9434220, Longitude: −43.1772478). Plastic waste, such as disposable cups and bags, was collected from each beach using sterile gloves to prevent contamination. Some waste showed signs of degradation, while others were covered with algae (Fig. 2). Using tweezers and scissors previously sterilized with 70% ethanol, smaller fragments of the waste were placed into sterile 15 mL Falcon tubes and stored in refrigerated containers (0–4 °C) to preserve the biofilms until arrival at the laboratory, where they were transferred to a freezer (−20 °C) until analysis.

### Sample processing

Before analysis, samples were thawed and gently rinsed with sterile distilled water to remove surface residues. From each sample, one fragment was aseptically collected to analyze its polymer chemical composition. Three other fragments were taken for molecular analysis in triplicate. Each fragment was placed in an Eppendorf tube, resulting in three subsamples per waste type (technical replicates) and a total of 18 samples for molecular analysis.

### Polymer composition

The samples were analyzed for their polymer composition using the methodology described in Castro et al. [21], employing a PerkinElmer infrared spectrometer (model Spectrum 3) coupled to the Spotlight 200i microscope (also from PerkinElmer) at the Chemistry Institute of UERJ. The analysis was conducted in attenuated total reflectance (ATR) mode with a Germanium (Ge) crystal, with a wavenumber range of 4000–600 cm⁻¹ and scans equal to 16. Analyses were performed using a Zinc Selenide (ZnSe) slide as a support medium. PerkinElmer Polymers Library was used to validate the results.

### Bacterial community analysis

The fragments intended for molecular analysis from the different samples were cut into tiny pieces and immersed in liquid nitrogen within a mortar for freezing. During the freezing process, these fragments were pulverized with a pestle. 0.5 g of the material from this procedure was transferred to tubes containing glass beads, provided in the Qiagen extraction kit.

DNA extraction was performed using the “PowerSoil Isolation Kit” (©Qiagen USA - www.qiagen.com.br) following the manufacturer’s instructions. DNA quality and concentration were evaluated by fluorimetry using a Qubit fluorometer (ThermoFisher - USA). For sequencing purposes, the variable V4-V5 region of the 16 S rRNA gene was targeted and amplified using the primers 515 F and 909R [22]. The amplicons were sequenced using the Illumina MiSeq platform (Illumina, San Diego, CA, USA) by StarSeq GmbH (Mainz, Germany).

After sequencing, the generated reads were processed using bioinformatics software. Subsequent analyses, including diversity, taxonomy, and classification, were conducted using RStudio software.

The raw data were demultiplexed and imported into the QIIME2 pipeline [23]. The DADA2 plugin [24] was utilized to assess read quality and cluster them into Amplicon Sequence Variants (ASVs). The taxonomic classification of each ASV was accomplished using the feature-classifier plugin with a Bayesian classifier trained with the SILVA 138 database [25]. Singletons, ASVs classified as Eukaryota and Archaea, off-targets, and organellar DNA (chloroplast and mitochondria) were discarded. Rarefaction curves were generated to evaluate the adequacy of sequencing depth.

### Statistical analysis

Alpha diversity analysis, including observed ASVs and Shannon index [26], was calculated using the R programming language (version 3.6.3) [27].

For beta diversity analysis, which examined variations in sample composition between plastic polymer type, multidimensional scaling (MDS) was employed [28], using the Phyloseq package [29]. R software generated an ordering among all communities through the Bray-Curtis distance. The differences between the bacterial profiles identified in each sample type were analyzed using the t-test and PERMANOVA tests, assuming *p* < 0.05 [30], after averaging the technical replicates per site for each polymer type.

### Estimation of potentially pathogenic bacteria

The identified bacterial genera were compared with a set of 292 genera potentially associated with human diseases (BGPH) and 72 genera potentially linked to diseases in marine organisms (BGPMO), as reported by Magalhães et al. [31] and Jurelevicius et al. [32]. For beta diversity analysis, which examined variations in sample composition between plastic types, multidimensional scaling (MDS) was employed by Wickelmaier [28], using the Phyloseq package [29].

## Results

### Polymer composition

**Fig. 3:**
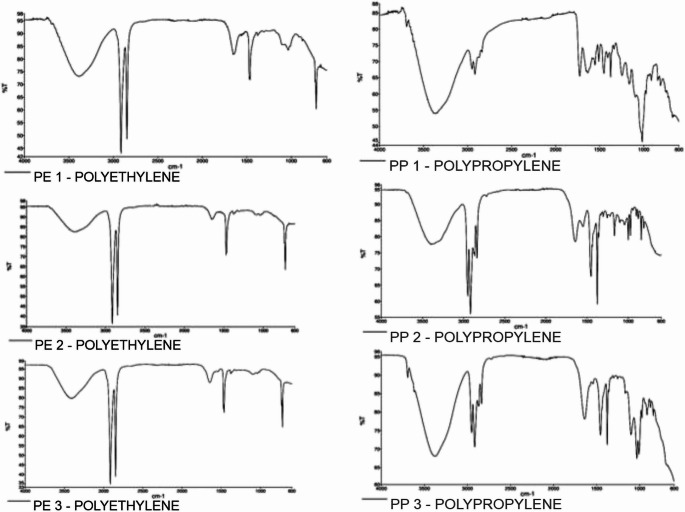
Image with the spectra resulting from the FTIR-ATR analyses. On the left from top to bottom, samples PE1, PE2, and PE3 (polyethylene), respectively, and on the right, PP1, PP2, and PP3 (polypropylene) in the same order

FTIR analysis revealed that the spectra of plastic bags are similar, indicating that they originate from the same polymer. The presence of absorption bands only for the CH2 group (2917, 2847, 1471, and 720 cm⁻¹) suggests that the polymer was polyethylene (PE). Plastic cups also exhibited similar spectra, corresponding to polypropylene (PP). Figure 3 shows the spectra acquired for each sample after analysis.

### The bacterial community associated with the samples

**Fig. 4:**
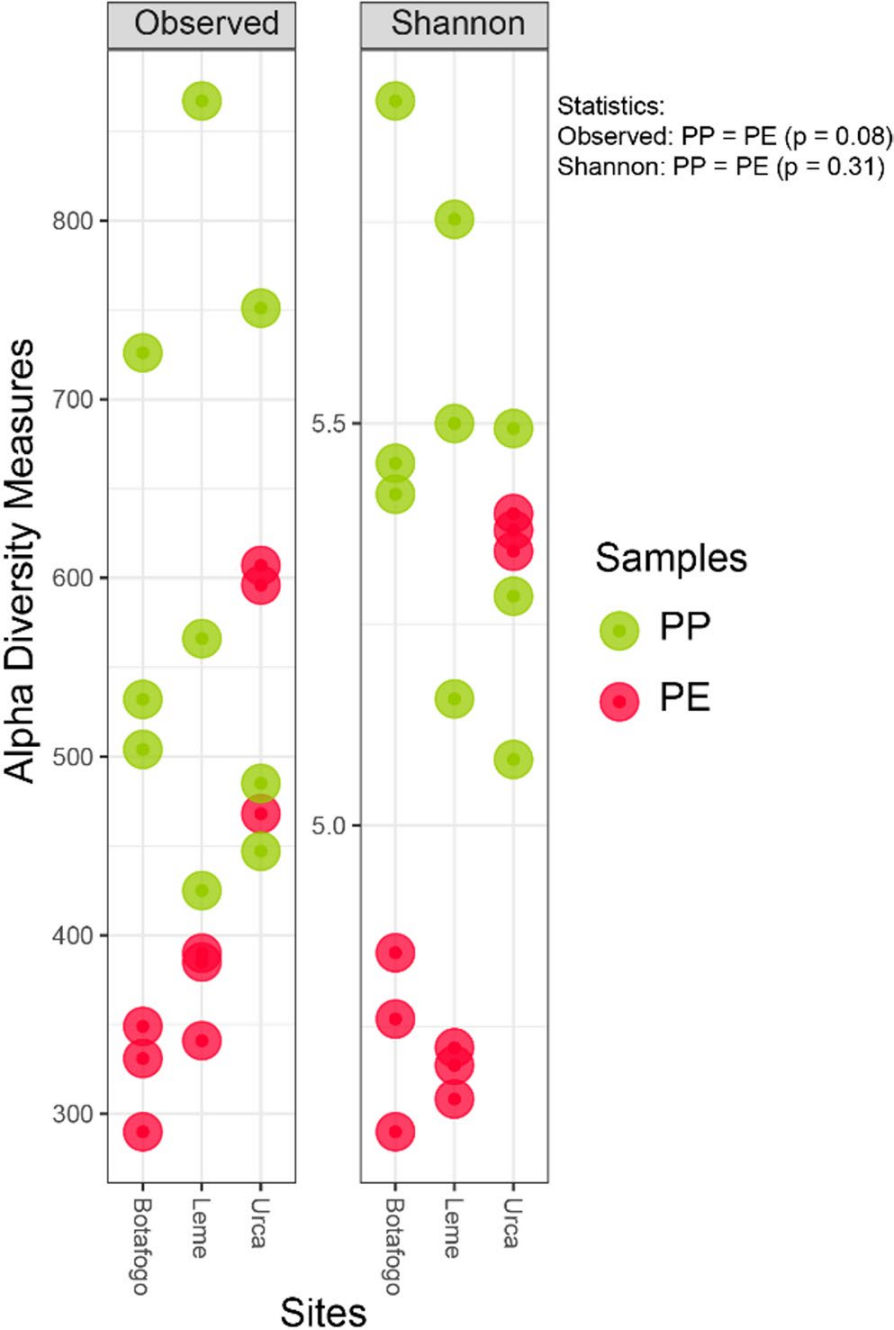
Alpha diversity plot showing the richness and diversity of the microbial community present in the plastic bag (PE) and plastic cup (PP) samples from each beach (left side). In the graph, individual samples of PE (red) and PP (green) refer to the richness and diversity of the microbial community on each beach

The richness and diversity of the bacterial community found in the samples are illustrated in Fig. 4. The “Observed” section of Fig. 4 illustrates the microbial richness within the plastic waste samples from each beach. The community diversity in the Plastisphere is depicted in the “Shannon” section. These graphs reveal that PP samples exhibit the same richness (t-test = 2.35, *p* = 0.08) and diversity (t-test = 1.15, *p* = 0.31) as PE.

**Fig. 5:**
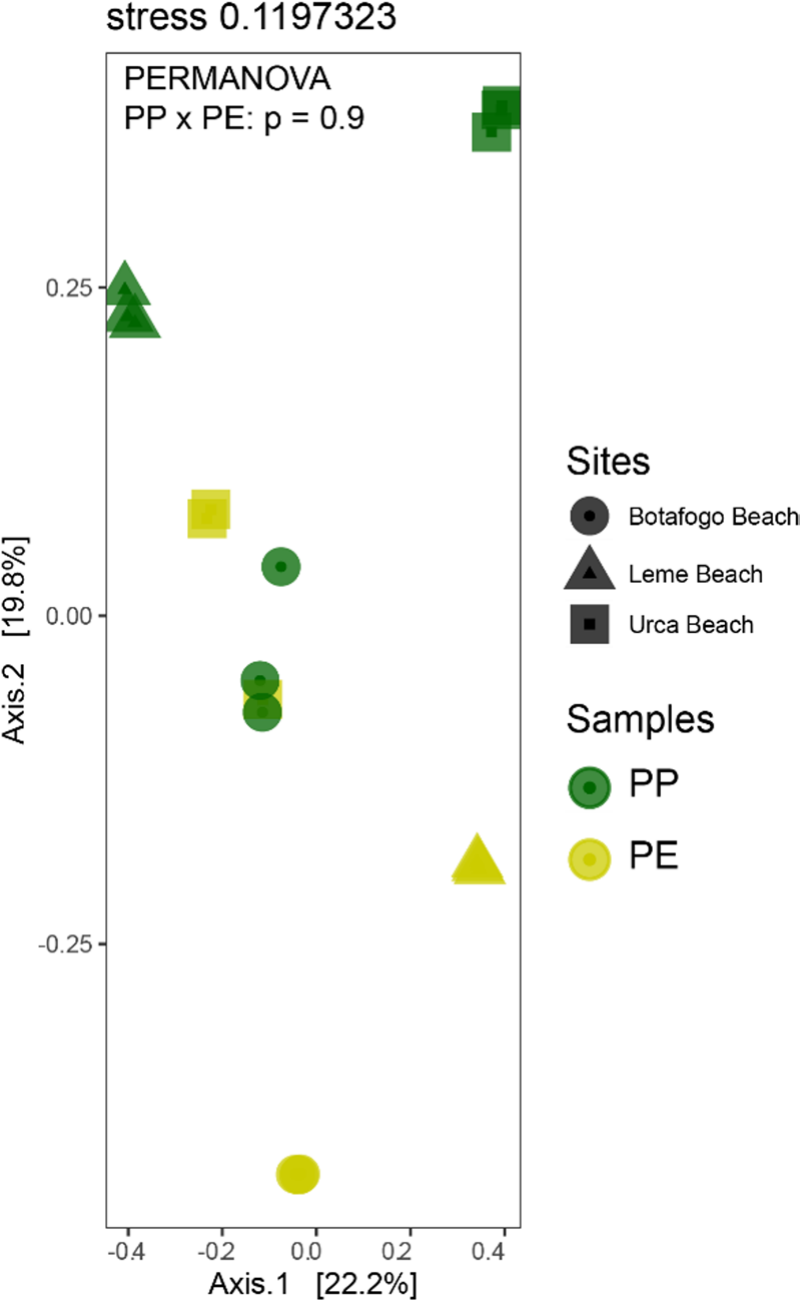
Non-metric multidimensional scaling (nMDS), where the profile of the plastic samples is evaluated through the classification by location and polymer composition. The colors green and yellow represent, respectively, polypropylene (PP) and polyethylene (PE). The symbols circle, triangle, and square are the collection sites

The nMDS analysis (Fig. 5) illustrates the distribution of bacterial profiles from plastic samples, categorized by polymer composition. The two primary axes explain 22.2% (Axis 1) and 19.8% (Axis 2) of the total variance, with a stress value of 0.119, indicating a good fit of the data in the two-dimensional space. Even though the figure indicates spatial separation among PP and PE, PERMANOVA indicates no statistical difference (F = 0.58, *p* = 0.9).

**Fig. 6:**
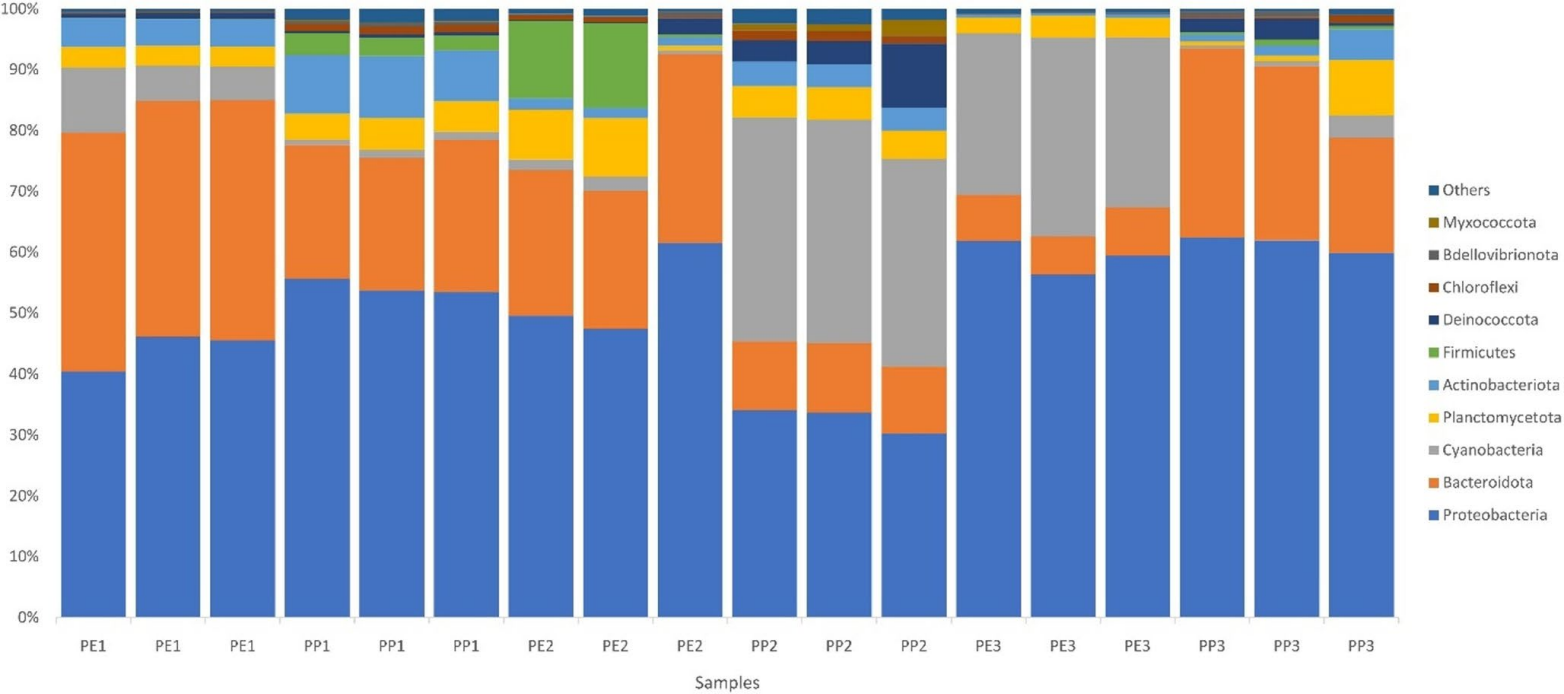
Relative abundance of microbial phyla found in plastic waste. The sample identification represents the polymer composition under analysis (PE for polyethylene and PP for polypropylene), followed by the beach from which the samples were collected (1- Leme, 2- Urca, 3- Botafogo)

Figure 6 displays the microbial phyla found in plastic waste, showing their abundance at each location and polymer type. Proteobacteria were predominant at all collection sites, particularly in Leme (PE1 and PP1) and Botafogo Beaches (PE3 and PP3). Bacteroidota was the second most abundant phylum, with a higher prevalence observed in the samples from Urca (PE2 and PP2). Cyanobacteria were prominent in PE3 and PP2. Planctomycetota appeared at all collection sites and polymer types with moderate but consistent abundance. Actinobacteriota were more evident in PP1, while Firmicutes and Deinococcota were more prominent in PE2 (Fig. 6). Although not very visible or abundant, it is noteworthy that Bdellovibrionota were present in both polymers, PE and PP, varying in abundance across different collection sites, being more noticeable in PP3, PP1, and PE2.

**Fig. 7:**
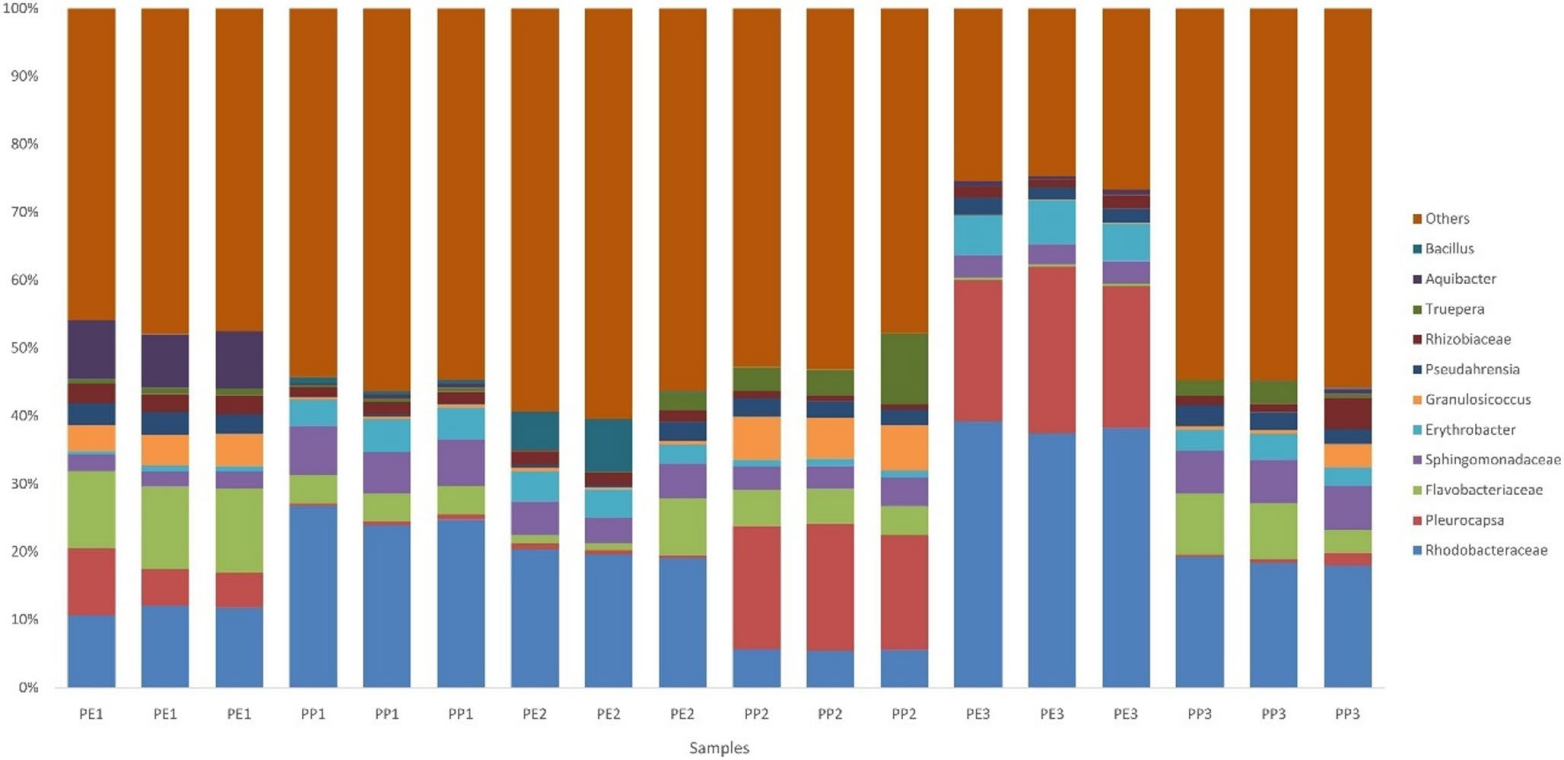
Relative abundance of the microbial taxonomic groups (genus and family level) at the different collection sites and waste types. The sample identification represents the polymer composition under analysis (PE for polyethylene and PP for polypropylene), followed by the beach from which the samples were collected (1- Leme, 2- Urca, 3- Botafogo)

Figure 7 depicts the relative abundance at the genus and family levels for each plastic waste sample and collection site. The category labeled "Other" in the graph includes various other genera identified in the analysis, albeit in smaller proportions (< 2%). 

The family Rhodobacteraceae is the most abundant in nearly all samples, with a particularly notable prevalence in PE3 and PP1. The genus *Pleurocapsa* is prominent in PE3 and PP2. Flavobacteriaceae and Sphingomonadaceae are present, but their abundance depends on the collection site and type of plastic. *Bacillus*,* Aquibacter*,* Truepera*,* Rhizobiaceae*,* Pseudahrensia*,* Granulosicoccus*,* Erythrobacter*, and other families are also present, but their abundances vary widely depending on the location and type of plastic polymer.

**Fig. 8:**
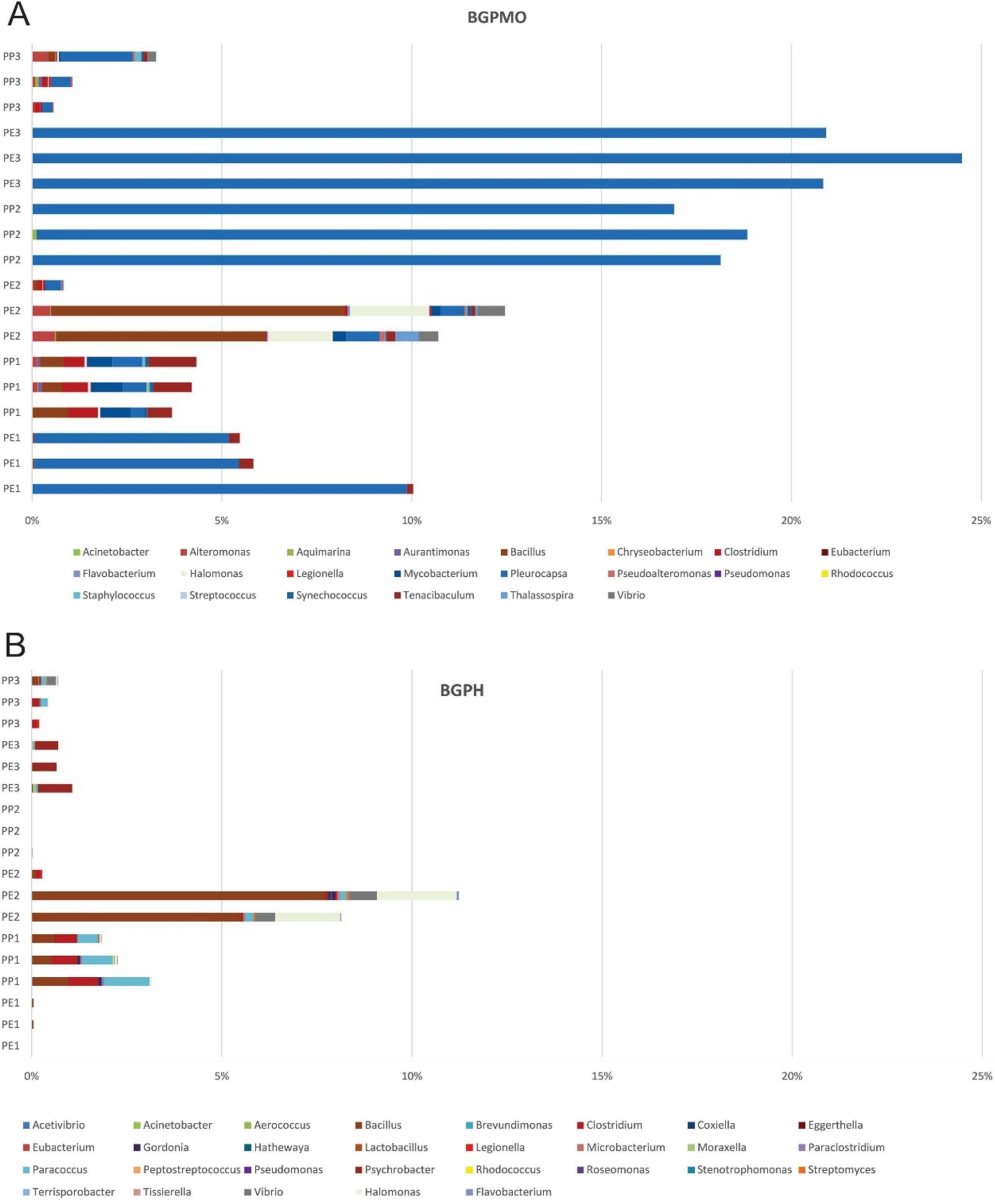
Potentially pathogenic bacteria for marine organisms (BGPMO), humans (BGPH), and abundances identified in the different plastic waste samples collected from the three studied beaches. The sample identification represents the polymer composition under analysis (PE for polyethylene and PP for polypropylene), followed by the beach from which the samples were collected (1- Leme, 2- Urca, 3- Botafogo)

Figure 8 shows the genera of bacteria potentially pathogenic to humans (BGPH) and marine organisms (BGPMO) found in the different plastic waste samples collected from the three beaches. BGPH was detected in all samples, except in PP2, but always in smaller proportions than BGPMO, which was present in all studied communities. While *Bacillus* was the prevalent genus found in PE2 and PP1, *Clostridium* and *Paracoccus* (also in PP1), *Psychrobacter* (in PE3), and *Halomonas* and *Vibrio* (in PE2) were other predominant genera in the BGPH group (Fig. 8A).

Among the BGPMO, *Pleurocapsa* was observed in all samples, with high abundance (> 15%) in PP2 and PE3. *Bacillus* (PE2 and PP1), *Tenacibaculum* (PP1 and PE1), *Halomonas* and *Vibrio* (PE2), and *Mycobacterium* and *Clostridium* (PP1) were other notable genera in some samples (Fig. 8B).

The greatest diversity of pathogens for humans and marine organisms was found in the samples PP1 and PE2.

## Discussion

Among the various threats of plastic pollution is the dispersal of pathogens and antimicrobial resistance genes through the plastisphere [33, 34]. Since the initial studies on this community, various marine environments have been investigated [3], although few have focused on tropical regions, where many areas lack adequate sanitation infrastructure and waters are contaminated by domestic effluents [4]. Two of the beaches in this study (Botafogo and Urca) are located along the waters of Guanabara Bay, where Silva et al. [4] isolated strains of *Vibrio* spp. and enteropathogenic *Escherichia coli*, while Leme, the other beach studied, is an oceanic beach near the entrance of this bay. These beaches, like others in the municipality of Rio de Janeiro, have been studied for the presence of solid waste [35, 36], but never in characterizing the microbial community associated with this waste.

The plastic cup and plastic bag samples collected from these beaches were identified as PP and PE, respectively. These two polymers are among the most collected plastics in marine environments [37, 38], likely due to their lower density compared to seawater, which allows them to float [39]. Additionally, these two types of plastic are among the most widely produced and consumed globally [40, 41].

The analysis of the plastisphere in plastic waste samples (PE and PP) revealed a diverse microbial community, with no evidence that polymer type influenced community composition. This finding corroborates the work of Zettler et al. [9], who also observed diverse bacterial communities in PE and PP samples, but differs from Amaral-Zettler et al. [42], who noted that these polymers influence the composition of the microbial communities found.

The slight differences in richness and diversity observed among the samples may be attributed to their exposure time to the ocean, as longer residence in the marine environment can directly influence community richness and diversity. Although present in all samples, the phylum Cyanobacteria was more abundant in PP2 and PE3. According to Zhai et al. [43], this phylum is primarily associated with the early stages of colonization in the plastisphere. On the other hand, the phylum Bacteroidota (especially Flavobacteriaceae) is considered a secondary colonizer [39]. Figure 6 clearly shows this succession process. In samples where Cyanobacteria predominated (PP2 and PE3), Bacteroidota abundance was lower. On the other hand, the opposite was observed in PE1 and PP3, indicating that the different samples are at distinct successional stages due to varying exposure times at their collection sites. The absence of differences in beta diversity between PP and PE suggests that their microbial communities were not shaped by plastic composition [20]. Rather, it indicates that colonization occurs during the dispersion of plastics from their source to the collection points.

The taxonomic groups found in plastic waste collected from the three beaches showed a predominance of microorganisms from the phylum Proteobacteria, commonly associated with plastic waste, surpassing their presence in seawater, glass, or organic surfaces [44], followed by Bacteroidota, with additional representation of Cyanobacteria, Planctomycetota, and Actinobacteriota. These findings align with previous studies [31, 45] showing that Proteobacteria and Bacteroidota are consistently dominant on microplastics in Brazilian and global marine environments. In Niterói beaches, also bathed by the waters of Guanabara Bay, Rhodobacteriaceae and Flavobacteriaceae were more abundant on microplastics than in the surrounding sand [31]. Similarly, research from Lagoa dos Patos, southern Brazil, reported a predominance of Proteobacteria on PP and PE samples, though with differences in other phyla, highlighting the strong environmental influence on plastisphere composition [45]. This influence is further reinforced by reviews of plastisphere studies conducted in the Northern Hemisphere [43].

Several of the dominant taxa detected are associated with metabolic capabilities relevant to plastic degradation or hydrocarbon processing. Rhodobacteraceae, a common marine family [46], occurred across all plastic types and sites and is frequently reported as a potential plastic degrader, along with other Alphaproteobacteria and Gammaproteobacteria [7, 47]. In our samples, Gammaproteobacteria—mainly *Granulosicoccus*—were particularly abundant in PP2 and PP3 and were also present in PE, consistent with observations from other plastisphere studies [47].

The family Sphingomonadaceae, commonly found across various polymers, including PE, PP, and polystyrene (PS) [44] and known for its ability to process hydrocarbons and produce carotenoids [48], was more abundant in PP but also present in PE. Additionally, *Erythrobacter*, a genus with phototrophic and aerobic capabilities and the ability to degrade PAHs [49, 50], was found across all polymers and sites. Another PAH-degrading genus, *Truepera*, was detected in all samples except PE3 and belongs to the phylum Deinococcota, also commonly found in plastisphere research [45, 51].

Within the family Flavobacteriaceae, the genus *Aquibacter* deserves attention due to its sensitivity to metal concentrations and its association with polluted environments [52]. Previously detected in metal-contaminated sediments from Sepetiba Bay, which faces issues similar to Guanabara Bay, Aquibacter’s elevated abundance in PE1 suggests that waste dispersal across areas with differing pollution levels may contribute to the microbial variation observed among sampling sites [53].

Although generally present in low abundance, several genera capable of causing disease in humans were detected in the plastisphere. *Bacillus*, which includes important pathogens such as *B. anthracis* and *B. cereus *[54], was the most notable, surpassing 5% in some samples. This low contribution differs from the results by Yang et al. [54], who reported *Bacillus* as one of the most abundant genera on marine microplastics and reflects the influence of factors such as location, plastic type, pollution level, and other environmental conditions on community structure. *Vibrio* and *Clostridium*, both containing clinically significant pathogenic species, contributed with ≤ 1% in abundance. Despite their low representation, their persistence on plastic surfaces raises public health concerns, especially considering previous reports of *Vibrio* spp. on microplastics in Guanabara Bay [4].

Multiple taxa with pathogenic potential to marine organisms were also identified. *Pleurocapsa*, known to cause disease in algae [55], was the most prominent, exceeding 20% in PE3 and PP2. Flavobacteriaceae, a family associated with coral disease and with disease states in sponges, starfish, macroalgae, and lobsters [56], occurred in nearly all samples and may additionally contribute to the spread of antibiotic-resistance genes [57, 58]. Rhodobacteraceae and Rhizobiaceae—both linked to coral diseases [48]—were also present in all samples, indicating that plastic waste may act as a vector for taxa harmful to coral ecosystems. While no corals occur in the study area, the spread of plastic waste could affect nearby coral-rich regions such as Arraial do Cabo and Angra dos Reis. Other genera of concern include *Bacillus*, which contains species that cause infections in fish and invertebrates [54, 59], and *Halomonas* and *Tenacibaculum*, each exceeding 1% in some samples (PE2 and PP1, respectively).

Planctomycetota, Actinobacteriota, Chloroflexi, and predatory taxa such as Bdellovibrionota and Myxococcota, were other phyla documented in this study, and all have been reported in plastisphere research [39].

Biofilm formation on plastics depends on factors such as polymer type, shape, exposure time, and environmental conditions, with polymer type being particularly well studied [54]. Carson et al. [60] reported higher bacterial abundance on PS compared to PE and PP, likely due to surface differences. Similarly, Zettler et al. [9] found distinct microbial communities on PP and PE, sharing less than 50% similarity. These authors reported that some microorganisms were more abundant on PP and high-density polyethylene (HDPE) surfaces than in the surrounding water and that the microbial communities on these plastics differed markedly from those in the ambient environment.

Our findings indicate that variations in microbial abundance across samples are more closely linked to the length of time the plastics remained in the marine environment. Although communities on different polymers formed separate clusters, no clear polymer-specific associations were observed, suggesting that exposure duration plays a stronger role in shaping microbial colonization. The presence of degraded plastics further supports prolonged environmental exposure and underscores the influence of durability on plastic pollution.

## Conclusion

The results of this study align with other research on the plastisphere [9, 15, 39, 44, 49], indicating a consistent presence of various microorganisms adhering to plastic surfaces. The bacteria identified in the samples are cited in the literature of other studies involving plastic and microplastic waste. Some observed genera include species involved in degradation and potential pathogens for animals and humans.

Although the samples analyzed showed no influence of the polymer composition on bacterial community formation, the exposure time of the waste in the sea appears to have a greater effect.

The gaps in our understanding create opportunities for future research to explore various aspects, such as the effects of microorganisms associated with plastics on marine life, the potential transfer of antimicrobial resistance, and the impacts on human health. Comparative analyses with the marine water microbial community can provide valuable context, helping to discern the unique attributes of the plastisphere.

Future studies investigating the genetic diversity and functional capabilities of microorganisms associated with plastic waste could provide insights into their roles in degradation and potential pathogenicity. Additionally, isolating and testing pathogenic bacteria and antimicrobial resistance genes would contribute important data on the health risks posed by the plastisphere.

By revealing the ecological consequences of plastic pollution and its potential implications for human health, our research contributes to the growing body of evidence highlighting the urgent need for effective mitigation strategies. This understanding benefits the local population and has global significance in addressing the widespread issue of marine pollution.

## References

[1] Our World in Data (2024) Plastic Pollution. Retrieved from https://ourworldindata.org/plastic-pollution. Accessed 07 July 2024

[2] Borrelle SB, Ringma J, Law KL, Monnahan CC, Lebreton L, McGivern A, Rochman CM (2020) Predicted growth in plastic waste exceeds efforts to mitigate plastic pollution. Science 369(6510):1515–1518. 10.1126/science.aba3656 32943526 10.1126/science.aba3656

[3] Jacquin J, Cheng J, Odobel C, Pandin C, Conan P, Pujo-Pay M, Barbe V, Meistertzheim AL, Ghiglione JF (2019) Microbial ecotoxicology of marine plastic debris: a review on colonization and biodegradation by the “Plastisphere.” Front Microbiol 10:865. 10.3389/fmicb.2019.00865 31073297 10.3389/fmicb.2019.00865 PMC6497127

[4] Silva MM, Maldonado GC, Castro RO, Felizardo JS, Cardoso RP, Anjos RM, Araújo FV (2019) Dispersal of potentially pathogenic bacteria by plastic debris in Guanbara Bay, RJ, Brazil. Mar Pollut Bull 141:561–568. 10.1016/j.marpolbul.2019.02.064 30955768 10.1016/j.marpolbul.2019.02.064

[5] Curren E, Leong SCY (2019) Profiles of bacterial assemblages from microplastics of tropical coastal environments. Sci Total Environ 655:313–320. 10.1016/j.scitotenv.2018.11.250 30471599 10.1016/j.scitotenv.2018.11.250

[6] Rodrigues A, Oliver DM, McCarron A, Quilliam RS (2019) Colonisation of plastic pellets (nurdles) by *E. coli* at public bathing beaches. Mar Pollut Bull 139:376–380. 10.1016/j.marpolbul.2019.01.011 30686440 10.1016/j.marpolbul.2019.01.011

[7] Almeida LRO, Ottoni JR, Passarini MRZ (2021) Plastics in the cold marine environment: a review on microbial biodegradation potential. Res. Soc. and Develop. 10(3):e49310313642. 10.33448/rsd-v10i3.13642

[8] Castro RO, Silva ML, Marques MRC, Araujo FV (2016) Evaluation of microplastics in Jurujuba Cove, Niteroi, RJ, Brazil, an area of mussel farming. Mar Pollut Bull 1:555–558. 10.1016/j.marpolbul.2016.05.037 10.1016/j.marpolbul.2016.05.037 27267118

[9] Zettler ER, Mincer TJ, Amaral-Zettler LA (2013) Life in the “Plastisphere”: microbial communities on plastic marine debris. Environ Sci Technol 47(13):7137–7146. 10.1021/es401288x 23745679 10.1021/es401288x

[10] Bergmann M, Gutow L, Klages M (2015) Marine anthropogenic litter. Springer Dordrecht Heidelberg, New York, London, p 456. 10.1007/978-3-319-16510-3

[11] Vethaak AD, Leslie HA (2016) Plastic debris is a human health issue. Environ Sci Technol 50:6825–6826. 10.1021/acs.est.6b02569 27331860 10.1021/acs.est.6b02569

[12] Artham T, Sudhakar M, Venkatesan R, Madhavan Nair C, Murty KVGK, Doble M (2009) Biofouling and stability of synthetic polymers in sea water. Int Biodeter Biodegrad 63(7):884–890. 10.1016/j.ibiod.2009.03.003

[13] Andrady AL (2011) Microplastics in the marine environment. Mar. Poll Bull 62:1596–1605. 10.1016/j.marpolbul.2011.05.030 10.1016/j.marpolbul.2011.05.030 21742351

[14] Kirstein IV, Wichels A, Gullans E, Krohne G, Gerdts G (2019) The Plastisphere – Uncovering tightly attached plastic specific microorganisms. PLoS ONE 14(4):e0215859. 10.1371/journal.pone.0215859 31013334 10.1371/journal.pone.0215859 PMC6478340

[15] Arias-Andres (2020) Who is where in the Plastisphere, and why does it matter? Mol. Ecol Resour 00:1–3. 10.1111/1755-0998.13161 10.1111/1755-0998.13161 32329966

[16] Dussud C, Hudec C, George M, Fabre P, Higgs P, Bruzaud S, Delort AM, Eyheraguibel B, Meistertzheim AL, Jacquin J, Cheng J, Callac N, Odobel C, Rabouille S, Ghiglione JF (2018) Colonization of Non-biodegradable and biodegradable plastics by marine microorganisms. Front Microbiol 9:1571. 10.3389/fmicb.2018.01571 30072962 10.3389/fmicb.2018.01571 PMC6058052

[17] Cresswell AJ, Baird D, Riddington R (2023) Microbial communities on marine plastics: implications for ecosystem function and plastic degradation. Sci Total Environ 891:164606

[18] Imran M, Das KR, Naik MM (2019) Co-selection of multi-antibiotic resistance in bacterial pathogens in metal and microplastic contaminated environments: an emerging health threat. Chemosp 215:846–857. 10.1016/j.chemosphere.2018.10.114 10.1016/j.chemosphere.2018.10.114 30359954

[19] Kaur K, Reddy S, Barathe P, Oak U, Shriram V, Kharat SS, Govarthanan M, Kumar V (2022) Microplastic-associated pathogens and antimicrobial resistance in environment. Chemosp 291(Pt 2):133005. 10.1016/j.chemosphere.2021.133005 10.1016/j.chemosphere.2021.133005 34813845

[20] INEA, Instituto Estadual do Ambiente do Rio de Janeiro (2024) https://www.inea.rj.gov.br/rio-de-janeiro/. Accessed 31 March 2022

[21] Castro RO, Silva ML, Marques MRC, Araújo FV (2020) Spatio-temporal evaluation of macro, meso and microplastics in surface waters, bottom and beach sediments of two embayments in Niterói. RJ Brazil Mar Poll Bull 160:111537. 10.1016/j.marpolbul.2020.111537 10.1016/j.marpolbul.2020.111537 32889507

[22] Apprill A, McNally S, Parsons R, Weber L (2015) Minor revision to V4 region SSU rRNA 806R gene primer greatly increases detection of SAR11 bacterioplankton. Aquat Microb Ecol. 10.3354/ame01753

[23] Kuczynski J, Stombaugh J, Walters WA, González A, Caporaso JG, Knight R (2012) Using QIIME to analyze 16S rRNA gene sequences from microbial communities. Curr Protoc Microbiol. Chapter 1:Unit 1E.5. 10.1002/9780471729259.mc01e05s27. PMID: 23184592; PMCID: PMC4477843 10.1002/9780471729259.mc01e05s27 PMC4477843 23184592

[24] Callahan BJ, McMurdie PJ, Rosen MJ, Han AW, Johnson AJ, Holmes SP (2016) DADA2: High-resolution sample inference from illumina amplicon data. Nat Methods 13(7):581–583. 10.1038/nmeth.3869 27214047 10.1038/nmeth.3869 PMC4927377

[25] Quast C, Pruesse E, Yilmaz P, Gerken J, Schweer T, Yarza P et al (2012) The ribosomal RNA gene database project: improved data processing and web-based tools. Nucleic Acids Res 41(D1):D590–D596. 10.1093/nar/23193283 PMC3531112

[26] Shade A (2017) Diversity is the question, not the answer. ISME J 11:1–6. 10.1038/ismej.2016.118 27636395 10.1038/ismej.2016.118 PMC5421358

[27] R Core Team (2015) A Language and environment for statistical computing. R Foundation for Statistical Computing, Vienna

[28] Wickelmaier F (2003) An introduction to MDS. Sound quality research unit. Aalborg Univ Denmark 46(5):26

[29] Mcmurdie PJ, Holmes S (2013) Phyloseq: an R package for reproducible interactive analysis and graphics of microbiome census data. PLoS One 8(4):e61217. 10.1371/journal.pone.0061217 23630581 10.1371/journal.pone.0061217 PMC3632530

[30] McKight PE, Najab J (2010) Kruskal-Wallis test. Corsini Encyclopedia Psychol 1:1–10. 10.1002/9780470479216.corpsy0491

[31] Magalhães EA, de Jesus HE, Pereira PHF, Gomes AS, Santos HF (2024) Beach sand plastispheres are hotspots for antibiotic resistance genes and potentially pathogenic bacteria even in beaches with good water quality. Environ Pollut 344:123237. 10.1016/j.envpol.2023.123237 38159625 10.1016/j.envpol.2023.123237

[32] Jurelevicius D, Cotta SR, Montezzi LF, Dias ACF, Mason OU, Picão RC (2021) Enrichment of potential pathogens in marine microbiomes with different degrees of anthropogenic activity. Environ Pollut 268:115757. 10.1016/j.envpol.2020.115757 33168375 10.1016/j.envpol.2020.115757

[33] Araujo FV, Castro RO, Silva ML, Silva MM (2021) Ecotoxicological effects of microplastics and associated pollutants. In: Kibenge FS, Baldisserotto B, Chong RS-M (eds) Aquaculture toxicology, vol v1. Academic Press, London, pp 189–227

[34] Taveira I, Castro RO, Cypriano J, Santos HF, Abreu F, Araújo FV (2024) Retrieving the real microbial diversity in aquatic plastisphere. Mar Pollut Bull 206:116719. 10.1016/j.marpolbul.2024.116719 39029147 10.1016/j.marpolbul.2024.116719

[35] Carvalho DG, Baptista Neto JA (2016) Microplastic pollution of the beaches of Guanabara Bay, Southeast Brazil. Ocean Coast Manage 128:10–17. 10.1016/j.ocecoaman.2016.04.009

[36] Abude RRS, Augusto M, Cardoso RS, Cabrini TMB (2021) Spatiotemporal variability of solid waste on sandy beaches with different access restrictions. Mar Pollut Bull 171:112743. 10.1016/j.marpolbul.2021.112743 34352534 10.1016/j.marpolbul.2021.112743

[37] Kirstein IV, Hensel F, Gomiero A, Iordachescua L, Vianello A, Wittgren HB, Vollertsena J (2021) Drinking plastics? – Quantification and qualification of microplastics in drinking water distribution systems by FTIR and Py-GCMS. Water Res 188:116519. 10.1016/j.watres.2020.116519 33091805 10.1016/j.watres.2020.116519

[38] Olivatto GP, Carreira R, Tornisielo VL, Montagner CC (2018) Microplásticos: contaminantes de Preocupação global no Antropoceno. Rev Virt De Quím 10(6):1968–1989. 10.21577/1984-6835.20180125

[39] Du Y, Liu X, Dong X, Yin Z (2022) A review on marine plastisphere: biodiversity, formation, and role in degradation. Comput Struct Biotechnol 20:975–988. 10.1016/j.csbj.2022.02.008 10.1016/j.csbj.2022.02.008 PMC8861569 35242288

[40] Lithner D, Larsson Å, Dave G (2011) Environmental and health hazard ranking and assessment of plastic polymers based on chemical composition. Sci Total Environ 409(18):3309–3324. 10.1016/j.scitotenv.2011.04.03810.1016/j.scitotenv.2011.04.038 21663944

[41] Achilias DS, Roupakias C, Megalokonomos P, Lappas AA, Antonakou ΕV (2007) Chemical recycling of plastic wastes made from polyethylene (LDPE and HDPE) and polypropylene (PP). Journ Haz Mat 149(3):536–542. 10.1016/j.jhazmat.2007.06.076 10.1016/j.jhazmat.2007.06.076 17681427

[42] Amaral-Zettler LA, Zettler ER, Mincer TJ (2020) Ecology of the plastisphere. Nat Rev Microbiol 18:139–151. 10.1038/s41579-019-0308-0 31937947 10.1038/s41579-019-0308-0

[43] Zhai X, Zhang XH, Yu M (2023) Microbial colonization and degradation of marine microplastics in the plastisphere: A review. Front Microbiol 14|. 10.3389/fmicb.2023.1127308 10.3389/fmicb.2023.1127308 PMC9981674 36876073

[44] Roager L, Sonnenschein EC (2019) Bacterial candidates for colonization and degradation of marine plastic debris. Environ Sci Technol 53:20, 11636–11643. 10.1021/acs.est.9b02212 31557003 10.1021/acs.est.9b02212

[45] Sérvulo T, Taylor JD, Proietti MC, Rodrigues LS, Puertas IP, Barutot RA, Lacerda ALF (2023) Plastisphere composition in a subtropical estuary: influence of season, incubation time and polymer type on plastic biofouling. Environ Pollut 332:121873. 10.1016/j.envpol.2023.121873 37244532 10.1016/j.envpol.2023.121873

[46] Pohlner M, Dlugosch L, Wemheuer B, Mills H, Engelen B, Reese BK (2019) The majority of active rhodobacteraceae in marine sediments belong to uncultured genera: A molecular approach to link their distribution to environmental conditions. Front Microbiol 10:659. 10.3389/fmicb.2019.00659 31001232 10.3389/fmicb.2019.00659 PMC6454203

[47] Xu X, Wang S, Gao F, Li J, Zheng L, Sun C, He C, Wang Z, Qu L (2019) Marine microplastic-associated bacterial community succession in response to geography, exposure time, and plastic type in China’s coastal seawaters. Mar Pollut Bull 145:278–286. 10.1016/j.marpolbul.2019.05.036 31590788 10.1016/j.marpolbul.2019.05.036

[48] Becker CC, Brandt M, Miller CA, Apprill A (2022) Microbial bioindicators of stony coral tissue loss disease identified in corals and overlying waters using a rapid field-based sequencing approach. Environ Microbiol 24:1166–1182. 10.1111/1462-2920.15718 34431191 10.1111/1462-2920.15718

[49] Oberbeckmann S, Labrenz M (2019) Marine microbial assemblages on microplastics: Diversity, Adaptation, and role in degradation. Ann Rev Mar Sci 12(1). 10.1146/annurev-marine-010419-010633 10.1146/annurev-marine-010419-010633 31226027

[50] Koh J, Bairoliya S, Salta M, Cho ZT, Fong J, Neo M, Cragg S, Cao B (2023) Sediment-driven plastisphere community assembly on plastic debris in tropical coastal and marine environments. Environ Int 179:108153. 10.1016/j.envint.2023.108153 37607427 10.1016/j.envint.2023.108153

[51] Lassen C, Hansen SF, Magnusson K, Hartmann NB, Rehne Jensen P, Nielsen TG, Brinch A (2015) Microplastics: Occurrence, effects and sources of releases to the environment in Denmark. Danish Environmental Protection Agency

[52] Wright RJ, Langille MGI, Walker TR (2021) Food or just a free ride? A meta-analysis reveals the global diversity of the plastisphere. ISME J 15(3):789–806. 10.1038/s41396-020-00814-9 33139870 10.1038/s41396-020-00814-9 PMC8027867

[53] Moreira VA, Cravo-Laureau C, Carvalho ACB, Baldy A, Bidone ED, Sabadini-Santos E, Duran R (2023) Microbial indicators along a metallic contamination gradient in tropical coastal sediments. J Hazard Mater 443:130244. 10.1016/j.jhazmat.2022.130244 36327839 10.1016/j.jhazmat.2022.130244

[54] Yang Y, Liu W, Zhang Z, Grossart HP, Gadd GM (2020) Microplastics provide new microbial niches in aquatic environments. Appl Microbiol Biotechnol 104:6501–6511. 10.1007/s00253-020-10704-x 32500269 10.1007/s00253-020-10704-x PMC7347703

[55] Egan S, Fernandes ND, Kumar V, Gardiner M, Thomas T (2014) Bacterial pathogens, virulence mechanism and host defence in marine macroalgae. Environ Microbiol 16(4), 925–938. 10.1111/1462-2920.1228856Hudson 10.1111/1462-2920.12288 24112830

[56] Hudson J, Egan S (2022) Opportunistic diseases in marine eukaryotes: could Bacteroidota be the next threat to ocean life? Environ Microbiol 24:4505–4518. 10.1111/1462-2920.1228856 35706128 10.1111/1462-2920.16094 PMC9804302

[57] Yang Y, Liu G, Song W, Ye C, Lin H, Li Z, Liu W (2019) Plastics in the marine environment are reservoirs for antibiotic and metal resistance genes. Environ Int 123:79–86. 10.1016/j.envint.2018.11.061 30502597 10.1016/j.envint.2018.11.061

[58] Zeng J, Pan Y, Yang J, Hou M, Zeng Z, Xiong W (2019) Metagenomic insights into the distribution of antibiotic resistome between the gut-associated. environments and the pristine environments. Environ Int 126:346–354. 10.1016/j.envint.2019.02.052 30826613 10.1016/j.envint.2019.02.052

[59] Tuipulotu DE, Mathur A, Ngo C, Man SM (2020) *Bacillus cereus*: epidemiology, virulence factors, and host–pathogen interactions. Trends Microbiol 29(5):458–471. 10.1016/j.tim.2020.09.003 33004259 10.1016/j.tim.2020.09.003

[60] Carson HS, Nerheim MS, Carroll KA, Eriksen M (2013) The plastic-associated microorganisms of the North Pacific Gyre. Mar Pollut Bull 15(1–2):126–132. 10.1016/j.marpolbul.2013.07.054 10.1016/j.marpolbul.2013.07.054 23993070

